# Supplementary material for: Assessment of GGE, AMMI, Regression, and Its Deviation Model to Identify Stable Rice Hybrids in Bangladesh
Source: Plants (Basel). 2022 Sep 7;11(18):2336. doi: 10.3390/plants11182336 (PMC9504418; doi:10.3390/plants11182336)
Supplement: Supplementary file 1 [file plants-11-02336-s001.zip › plants-1891844-supplementary.pdf]

Supplementary Table S1. Temperature, relative humidity and rainfall during the growing period of twenty six promising hybrid rice genotypes

Gazipur

Month	Temperature (°C)		Relative humidity (%)	Rainfall (mm)
	Maximum	Minimum		
December-2019	24.84	14.622	74.75	3.6
January-2020	24.34	13.60	76.36	0.9
February-2020	28.0	14.52	62.63	0.02
March-2020	32.7	19.63	56.23	0.53
April-2020	33.57	22.66	64.6	4.26
May-2020	33.70	24.33	71.09	9.18

Source: BRRI weather report, Gazipur, 2019-2020
Agro-ecological zone (AEZ): Madhupur Tract; Latitude- 23º48′37″ N, Longitude-90º24′45″ E

Barisal

Month	Temperature (°C)		Relative humidity (%)	Rainfall (mm)
	Maximum	Minimum		
December-2019	24.31	13.89	74.24	0.45
January-2020	23.04	12.86	76.29	1.88
February-2020	26.71	13.29	66.18	0.00
March-2020	32.71	19.88	66.11	0.16
April-2020	32.89	22.53	73.63	5.57
May-2020	32.58	24.92	78.38	8.07

Source: BRRI weather report, Barisal, 2019-2020
AEZ: Ganges Tidal Floodplain; Latitude-22º42′36″ N, Longitude-90º21′12″ E

Iswardi

Month	Temperature (°C)		Relative humidity (%)	Rainfall (mm)
	Maximum	Minimum		
December-2019	34.17	12.35	80.18	0.19
January-2020	34.03	11.29	80.72	0.74
February-2020	33.81	12.20	73.43	0.00
March-2020	33.93	17.77	70.35	0.68
April-2020	33.84	21.7	70.23	0.97
May-2020	32.65	24.08	79.19	12.26

Source: BRRI weather report, Iswardi, 2019-2020
AEZ: Active Brahmaputra Jamuna Floodplain; Latitude-24º0′46″ N, Longitude-89º15′32″ E
